# The effects of resveratrol in animal models of primary osteoporosis: a systematic review and meta-analysis

**DOI:** 10.1186/s13018-024-04595-1

**Published:** 2024-02-13

**Authors:** Rongxian An, Qian Luo, Lei Li, Dinglu Cui, Jingchun Jin

**Affiliations:** 1 Yanbian University Hospital, Yanji, China; 2 Baoji Traditional Chinese Medicine Hospital, Baoji, China

**Keywords:** Primary osteoporosis, Resveratrol, Animal models

## Abstract

**Background:**

There is still a lack of sufficient evidence-based medical data on the effect of resveratrol (Res) on primary osteoporosis (OP). This meta-analysis aimed to comprehensively evaluate the role of Res in animal models of primary OP.

**Methods:**

The PubMed, Cochrane Library, Web of Science and Embase databases were searched up to August 2023. The risk of bias was assessed by the SYRCLE RoB tool. Random- or fixed-effects models were used to determine the 90% confidence interval (CI) or standardized mean difference (SMD). Statistical analysis was performed with RevMan 5.4 and Stata 14.0.

**Results:**

A total of 24 studies containing 714 individuals were included. Compared with those in the control group, the bone mineral density (BMD) (*P* < 0.00001), bone volume/total volume (BV/TV) (*P* < 0.001), trabecular thickness (Tb.Th) (*P* < 0.00001), and trabecular number (Tb.N) (*P* < 0.00001) were markedly greater, and the trabecular separation (Tb.Sp) (*P* < 0.00001) was significantly greater. Compared with the control group, the Res group also exhibited marked decreases in alkaline phosphatase (ALP) (*P* < 0.05), tartrate-resistant acid phosphatase 5b (TRAP5b) (*P* < 0.01), and type I collagen strong carboxyl peptide (CTX-1) (*P* < 0.00001) and a marked increase in osteoprotegerin (OPG) (*P* < 0.00001).

**Conclusion:**

In summary, we concluded that Res can markedly increase BMD, improve morphometric indices of trabecular microstructure and serum bone turnover markers (BTMs), and exert a protective effect in animal models of primary osteoporosis. This study can supply experimental reference for Res in primary osteoporosis treatment.

**Supplementary Information:**

The online version contains supplementary material available at 10.1186/s13018-024-04595-1.

## Introduction

Osteoporosis (OP) is a systemic bone disease characterized by damage to the bone microstructure and decreased bone mass, resulting in bone fragility and easy fracture [1, 2]. Primary osteoporosis, as a major part of OP, is currently a major public health problem facing patients and medical practitioners globally. A decreasing BMD not only increases the incidence of fractures but also has an incalculable impact on patients' financial status and personal and even whole-family quality of life, given that most patients with primary OP are elderly patients (postmenopausal OP and senile OP) [3, 4]. Clinically, anti-OP drugs are categorized into anti-absorptive and pro-synthetic drugs. Widely used options include bisphosphonates (BP), selective estrogen receptor modulators (SERM), and RANK-ligand inhibitors. Despite their popularity, these drugs are associated with various adverse effects. For example, Denosumab, a type of BP, significantly increases BMD in the spine and hip of postmenopausal women with OP. However, due to severe gastrointestinal reactions such as acid reflux, nausea, and vomiting, many patients opt for intravenous administration over oral, potentially impacting treatment compliance of patients [5–8]. For another example, although oestrogen replacement treatment has a significant effect on treating postmenopausal OP [9], studies have shown that this therapy may increase the risk of breast and uterine cancer. Thus, there is a critical need to identify drugs that are more effective, convenient, and safer for primary OP.

Resveratrol (Res) is a polyphenolic phytoestrogen that is present in the skin of red grapes, peanuts and various other fruits [10, 11] and has potent antioxidant, anti-inflammatory, antiageing, neuroprotective, anticarcinogenic and cardioprotective effects [12, 13]. In vitro evidence has shown that Res can improve the activity of osteoblasts and inhibit the differentiation of osteoclasts [14, 15]. For example, in vitro, Res increases ALP in a dose-dependent manner by promoting the differentiation of osteoblasts [16]. In vivo studies have shown that Res can improve BMD and prevent bone loss in young rats subjected to tail suspension, in ovariectomized (OVX) rats and in old rats subjected to hind limb suspension [17–19]. However, a large number of existing studies have not yet systematically summarized and analyzed the topic. Therefore, this review aims to comprehensively explore the role of Res in an animal model of primary OP through the use of a meta-analysis of animal experiments for the first time.

## Methods

The meta-analysis was performed in accordance with PRISMA guidelines (Additional file 1 ) and registered on the PROSPERO platform of the International Register of Systematic Evaluations (No. CRD42023478041).

### Data sources and search

Cochrane Library, PubMed, Web of Science and EMBASE were searched for studies of Res in animal models of primary OP up to August 2023.

### Study selection

The inclusion criteria were as follows: (1) The animal model was primary OP; (2) The animal models of primary OP were established by all kinds of methods, such as age-related OP, orchiectomy and ovariectomy; (3) The treatment group was given Res only, while the control group was given either no treatment or saline treatment; (4) The main results were bone mineral density (BMD); the second outcomes were morphometric indices of trabecular microstructure, including bone volume/total volume (BV/TV), trabecular number (Tb.N), trabecular thickness (Tb.Th), and trabecular separation (Tb.Sp); and serum bone turnover markers (BTMs), including osteocalcin (OC), alkaline phosphatase (ALP), serum osteoprotegerin (OPG), bone alkaline phosphatase (bALP), type I collagen strong carboxyl peptide (CTX-1), and tartrate-resistant acid phosphatase 5b (TRAP5b). The exclusion criteria were as follows: (1) reviews, cases, clinical trials, cell studies or other studies; (2) other animal models; and (3) other medicines.

### Data extraction

Two authors independently extracted the study characteristics (publication year, first author and sample size), method of modeling, basic characteristics, intervention, and outcome information. All the data were acquired, and several subgroup analyses were carried out for different dosages, modeling-established standards or patient positions. Disputes between the two radiologists were resolved by talking with a third person.

### The risk of bias assessment

The risk of bias of the included studies was evaluated by the SYRCLE risk of bias tool [20] (Fig. 2); the risk of bias was classified as “high”, “low” or “unclear”. Disagreements between An and Luo were resolved by Dr. Jin.

### Subgroup and sensitivity analysis

Because of the limited sample size, the number of female animals was greater than the number of male animals, the data on age and weight were incomplete, and the methods of modeling were different. In this review, a subgroup analysis was conducted even though it was difficult. If there was obvious heterogeneity in the primary outcome (*I*^2^ > 50%), this study was subjected to sensitivity analysis. Moreover, the stability of all outcomes was evaluated by ignoring each study in sequence.

### Data synthesis

Excel 2016, Stata 14.0, and RevMan 5.4 were used to perform this analysis. When the data were reported as the mean ± SEM (standard error of the mean), we transformed the SEM into the standard deviation (SD) using the formula ðSEM = SD/square root of the sample sizeÞ to avoid obfuscating the distinctive usage between the SD and SEM. Statistical heterogeneity was assessed by the chi-square test and the I^2^ test. A fixed-effects model was selected if *I*^2^ was < 50%; otherwise, the random-effects model was selected. Several independent groups in a study (e.g., various doses) were considered separate datasets. *P* < 0.05 indicated statistical significance.

## Results

**Fig. 1:**
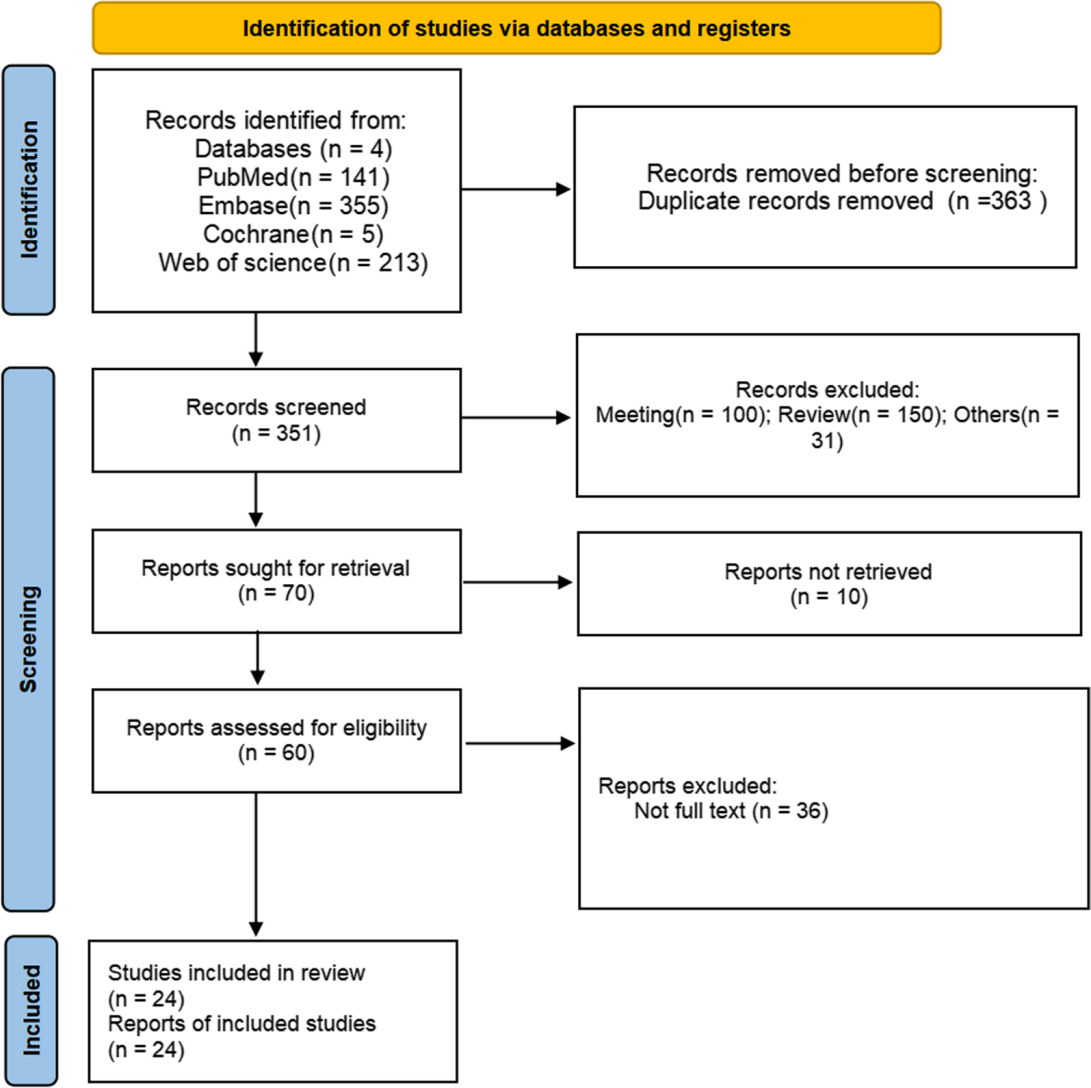
Flowchart of the literature search and selection process

A total of 714 studies were selected. After removing duplicates, 351 studies remained. Sixty studies were left for full-text screening after screening the titles and abstracts. Finally, 24 studies were analyzed. The basic characteristics of the final 24 studies [15, 17, 19, 21–41] are shown in Table 1. The search process is shown in Fig. 1.

**Table 1:** Characteristics of the included studies in the meta-analysis

Study	Species	Sex	Weight (g)	Age	N (T/no-T)	Model (establish; modeling standard)	Treatment group (administration;dose; course of treatment)	Outcome index
Chen [23]	Rat	Female	NA	略 NA	8/8	Ovariectomy	Two weeks after OVX procedures; intragastric administration of 0.2 μM RES once;about 10 weeks	Bmd
Jing Feng [25]	Rat	Female	280–350	3 3 months	8/8(low-dose)/8(middle)/8(high)	Ovariectomy	Orally administrated at the dosage of 5, 25and 45 mg/kg/d, respectively, 7 days after operation for 8 weeks	Bmd,BV/TV, Tb.Th,Tb.N, Tb.Sp, alp,oc
Omnia Ameen [21]	Rat	Male	350–400	18–20 months	10/10	Aging-dependent male osteoporosis	Receiving resveratrol; 20 mg/kg/day for 6 weeks	Balp
Wei Wan [34]	Rat	Female	250 ± 10	10–12 weeks	10/10(low-dose)/10(middle)/10(high)	Ovariectomy	Res dissolved in 5 ml of normal saline and administered at the dosage of 10, 20, and 40 mg/kg/d to rats intragastrically for 8 weeks	Bmd
Liwei Wei [36]	Rat	Female	NA	6 months	8/8	Ovariectomy	Two weeks after ovariectomy, rats in OVX and Res groups were received, respectively, Res solution at 10 mg/kg body weight by daily intraperitoneal injection or saline for 12 weeks	Bmd,BV/TV, Tb.Th,Tb.N, Tb.Sp, balp, pg, TRACP-5b, CTX-1
Alka Khera [28]	Rat	Female	NA	3 months	6/6	Ovariectomy	The diet was mixed with resveratrol (625 µg/Kg body weight/day) and administered orally to experimental animals as diet pellets for 4 weeks	Bmd
Zamai et al. [38]	Rat	Female	NA	4 months	10/10	Ovariectomy	The administration of Res (10 mg/Kg) and placebo was performed via gavage during all experiment period after ovariectomy surgery; 22 weeks	Tb.N,Tb.Sp, BV/TV
Qian Lin [29]	Rat	Female	254.91 ± 18.01	3 months	8/8	Ovariectomy	OVX rats with Res 5 mg, 15 mg, 45 mg × kgbw-1 × day-1, respectively, for 13 weeks	Bmd
Yan-Ling Feng [26]	Rat	Female	220 ± 19.27	3 months	10/10	Ovariectomy	RES solution (40 mg/kg body weight, once daily; 10 weeks	Bmd,BV/TV, Tb.Th,Tb.N, Tb.Sp
Elseweidy et al. [24]	Rat	Female	200–220	3 months	6/6	Ovariectomy	OVX rats that received 80 mg/kg/day of Res orally for 8 weeks (Res)	Bmd, CTX-1, alp
Yixuan Jiang [27]	Mice	Female	NA	8 weeks	4/4	Ovariectomy	RES (40 mg/kg body weight) was performed intraperitoneally once every day for 8 weeks	BV/TV,Tb.N, Tb.Sp, CTX-1
Liu et al. [17]	Rat	Female	220–250	NA	11/11	Ovariectomy	RES group treated with 0.7 mg/kg of body weight of resveratrol. Tested materials were given by gavage for 12 weeks after ovariectomy	Bmd
Haifeng Zhao [19]	Rat	Female	200–220	3–4 months	10/10(low-dose)/10(middle)/10(high)	Ovariectomy	Res(20, 40,,80 mg/kg/day) was orally administered, respectively, through a custom-made stomach tube for 12 weeks	Bmd,Tb.Th, Tb.N, Tb.Sp, alp
Lee et al. [15]	Rat	Male	NA	6 months	7/7	Ageing rats	20 mg/kg/day; 3 months	BV/TV,OC,alp, CTX-1
Tresguerres et al. [33]	Rat	Male	NA	22 months	10/10	Ageing rats	Treated with Res at dosages of 10 mg/kg per day; 10 weeks	BV/TV,Tb.Th, Tb.N,Tb.Sp, CTX-1, OC
Sehmisch et al. [32]	Rat	Female	220–260	3 months	11/11(low-dose)/11(high)	Ovariectomy	The rats received daily doses of 5 mg/kg bw for RES low and 50 mg/kg bw for RES high; 3 months	Bmd
Ye Zhang [39]	Rat	Female	NA	NA	8/8(low-dose)/8(middle)/8(high)	Ovariectomy	Given orally with RES (50, 100, and 200 mg/day); 12 weeks	Bmd,balp, opg, TRAP-5b, CTX-1, alp,BV/TV, Tb.Th, Tb.N, Tb.Sp
Yujin Zhang [40]	Rat	Female	220 ± 18	3 months	8/8(low-dose)/8(middle)/8(high)	Ovariectomy	RES-L, RES-M and RES-H treatment group were, respectively, given RES (dimethyl sulfoxide, DWSO), the concentration of DWSO in the solution was 0.5% of 5 mg/(kg.d), 15 mg/(kg.d) and 45 mg/(kg.d) by gavage; 12 weeks	Bmd,oc,alp, CTX-1
Wang(35)	Rat	Female	300	12 weeks	8/8(low-dose)/8(high)	Ovariectomy	Resveratrol(5 mg/kg/day)/ (45 mg/kg/day) was administered orally, respectively, to rats for 10 weeks	Bmd,BV/TV, Tb.Th,Tb.N, Tb.Sp, opg
Yuquan Shi[37]	Mice	Female	NA	8 weeks	8/8	Ovariectomy	The mice received treatment with Res (7 mg/kg) on the second day after OVX surgery; 6 weeks	BV/TV,Tb.Th, Tb.N,Tb.Sp, balp, TRAP-5b
Sakr et al. [31]	Rat	Male	300 ± 25	14 weeks	8/8	Orchiectomy	Res time-release pellets (50 mg, Cat. No. NX-999) were implanted subcutaneously (one pellet/animal) to release the whole concentration of Res over 90 days	Bmd,opg,TRAP-5b, OC, alp
Osturk et al. [30]	Rat	Female	200–250	3 months	12/12(low-dose)/12(high)	Ovariectomy	Res was administered by oral gavage (40 and 80 mg/kg/day) for ten weeks	Bmd, Alp, OC, BV/TV,Tb.Th, Tb.Sp
Zuozhong Liu [41]	Mice	Female	NA	8 weeks	6/6	Ovariectomy	The mice received treatment with Res (7 mg/kg) on the second day after OVX surgery; 6 weeks	BV/TV,Tb.Th, Tb.N, Tb.Sp
Basem [22]	Rat	Female	365 ± 10	12–14 weeks	10/10	Ovariectomy	45 µg/kg/day, orally by gavage; 16 weeks	Bmd,opg,TRAP-5b, OC, alp

## The risk of bias and publication bias

**Fig. 2:**
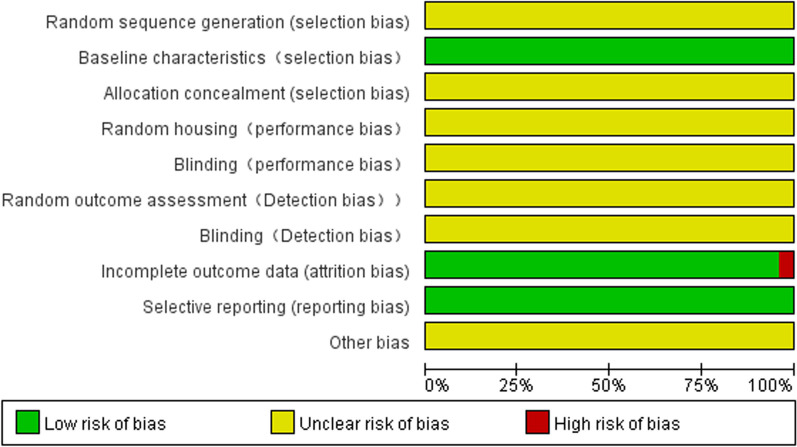
Risk-of-bias summary using the SYRCLE risk of bias tool

Several studies (Fig. 2) were thought to have an “unclear risk of bias”, for example, random sequence generation, random housing and random outcome assessment. A low risk of bias was observed for incomplete outcome data, baseline characteristics and selective reporting in all studies except one [19]. Moreover, the funnel plot (*n* > ten papers) showed that the stability of the results was not affected by publication bias (Additional file 1 ).

## Effectiveness

### Primary outcomes-BMD (Figs. 3 and 4)

**Fig. 3:**
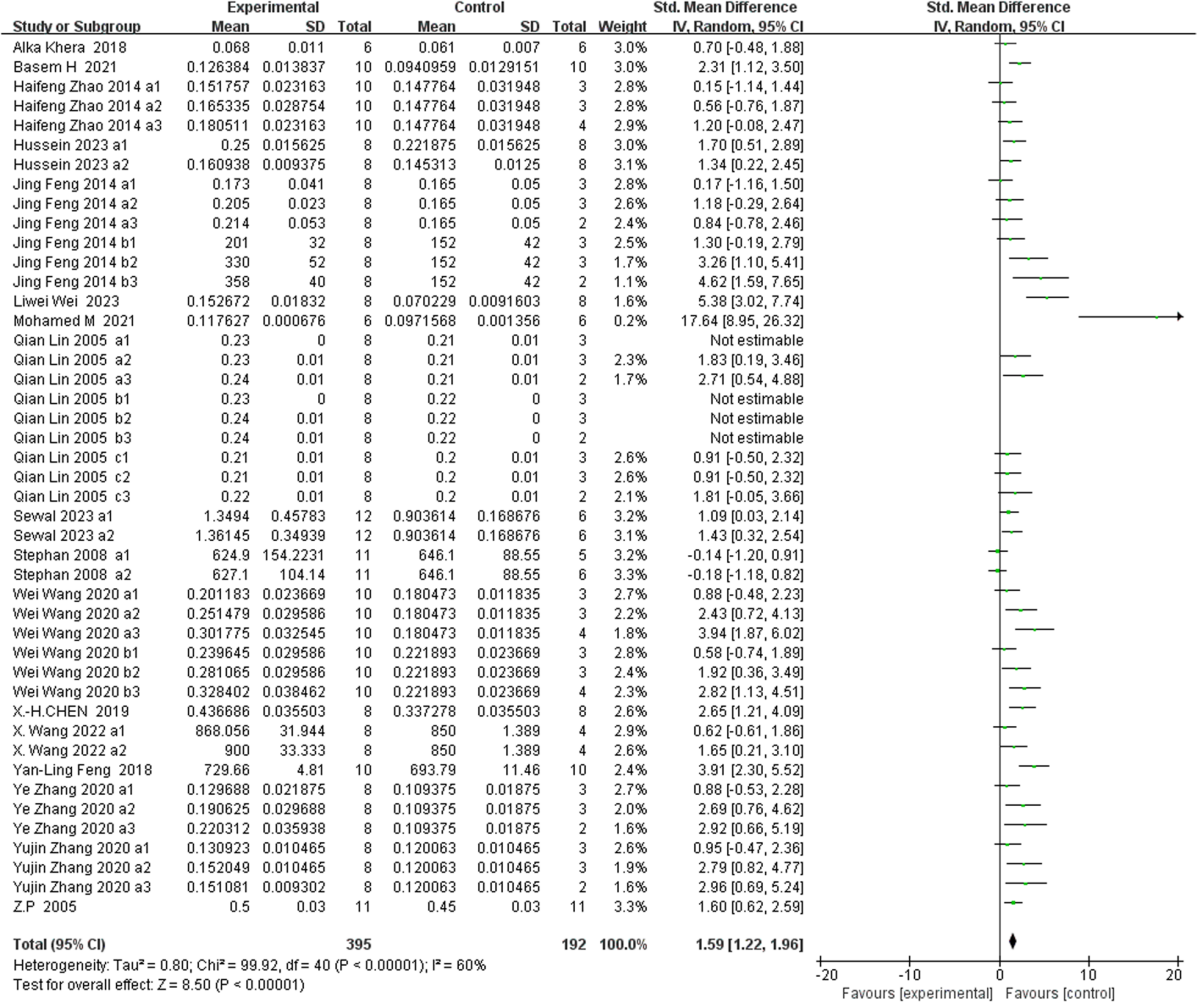
The meta-analysis results of the Res for BMD

**Fig. 4:**
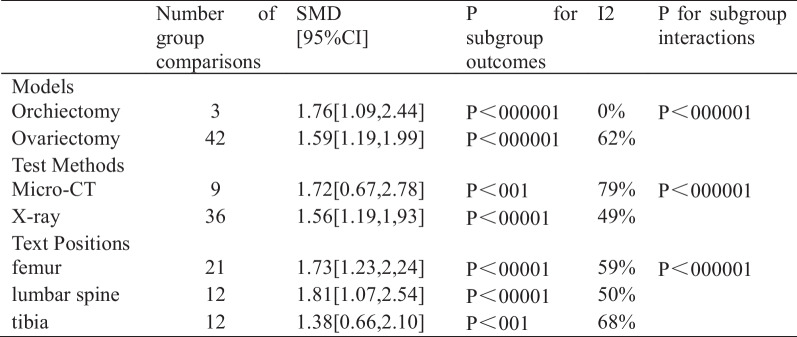
Subgroup analysis of Res for BMD

Analysis of 45 studies [17, 19, 23–26, 28–32, 34, 36, 39, 40] showed that, compared with the control group, the Res group had a markedly greater BMD (*n* = 587; SMD, 1.59; 95% confidence interval (CI), 1.22 to 1.96; I^2^ = 60%, *P* < 0.00001). Due to the high heterogeneity, we analyzed the BMD subgroups according to the methods of modeling, test methods and test positions (Fig. 4). Subgroup analysis according to the above several points showed no significant reduction in heterogeneity, which may remind us to search for other more suitable points (Additional file 1).

## Secondary outcomes

### Morphometric indices of the trabecular microstructure (Figs. 5, 6, 7, 8)

**Fig. 5:**
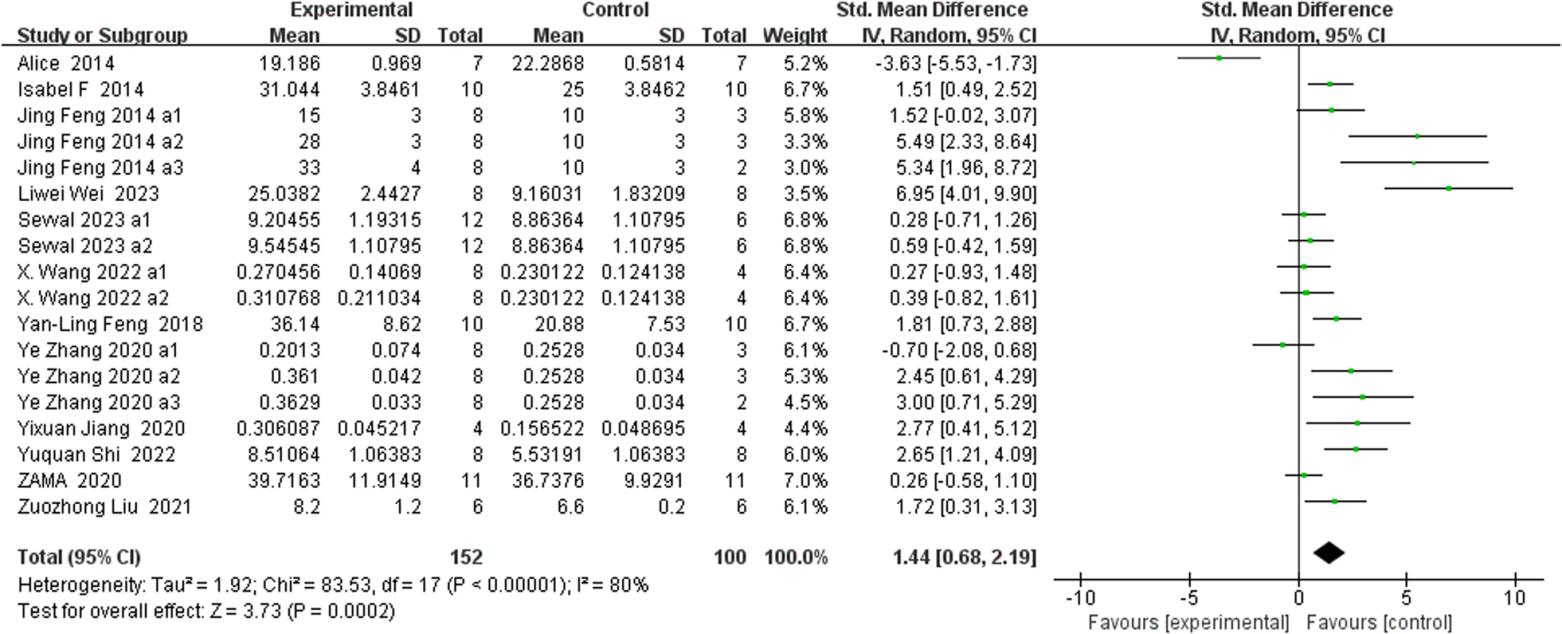
The meta-analysis results of Res for BV/TV

**Fig. 6:**
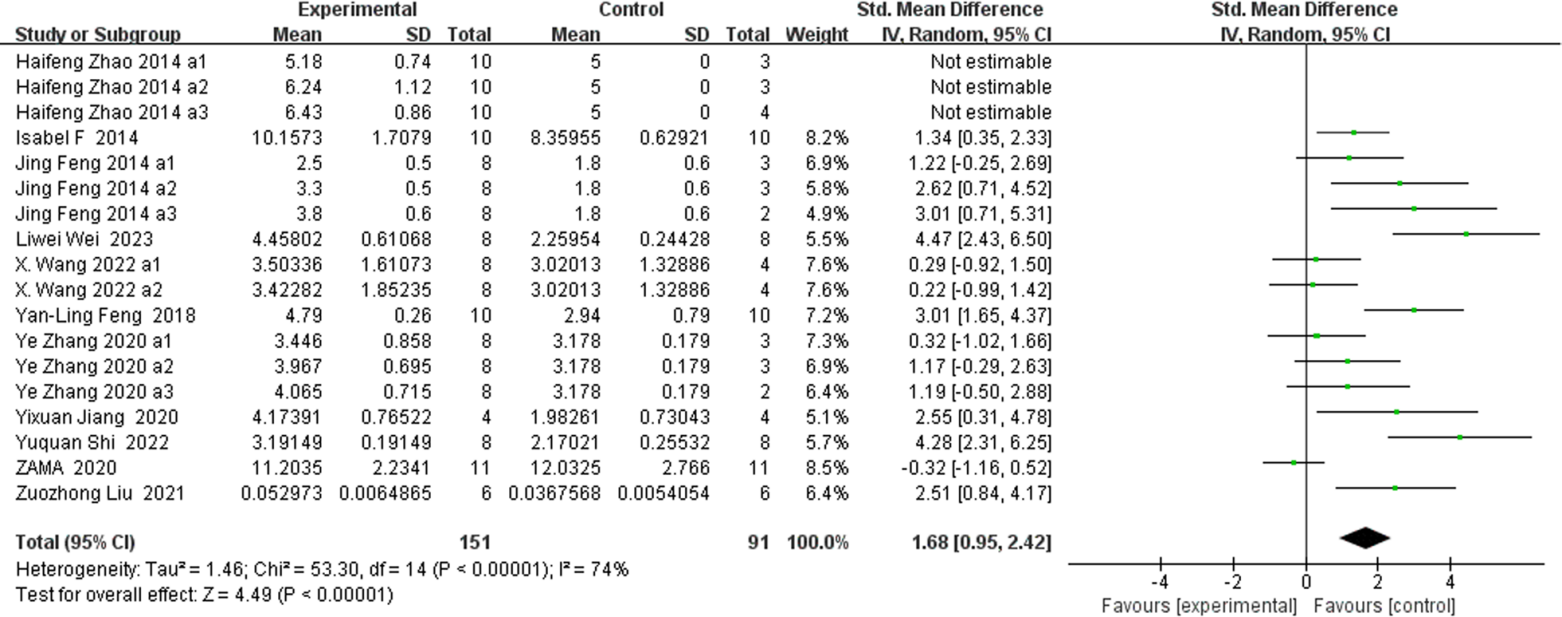
The meta-analysis results of Res for Tb.N

**Fig. 7:**
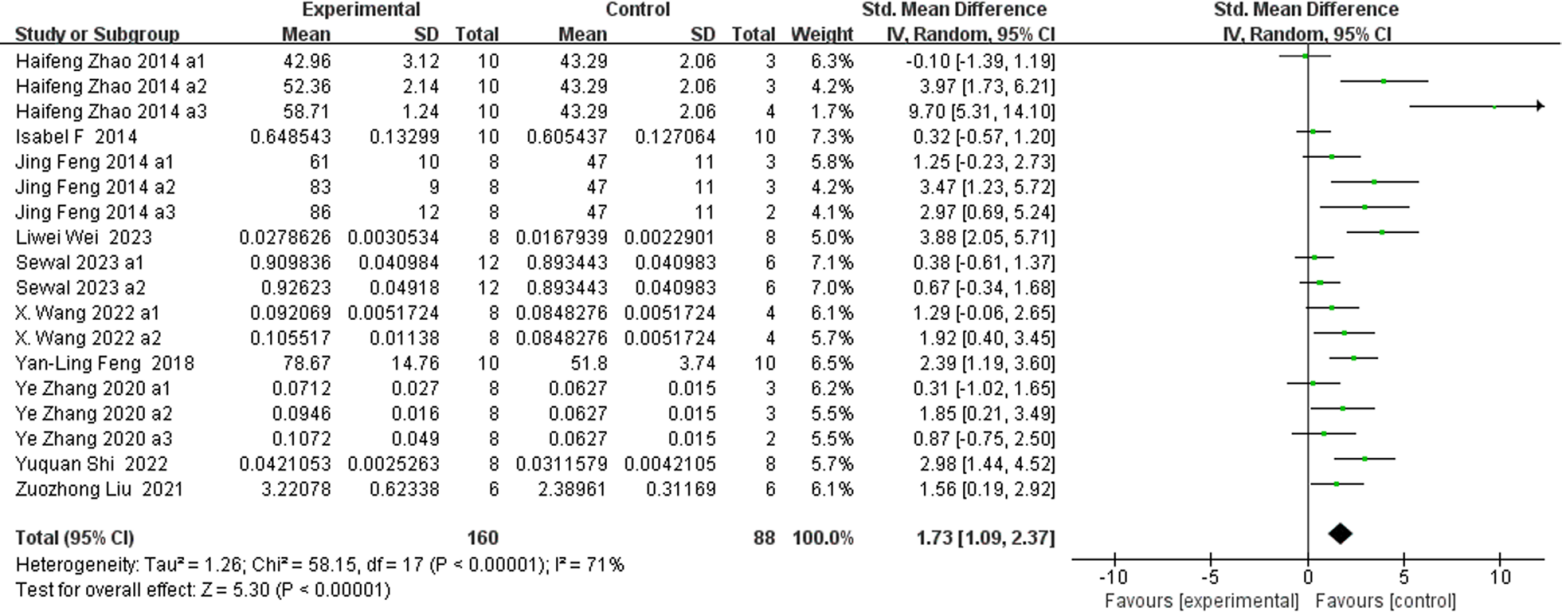
The meta-analysis results of Res for Tb.Th

**Fig. 8:**
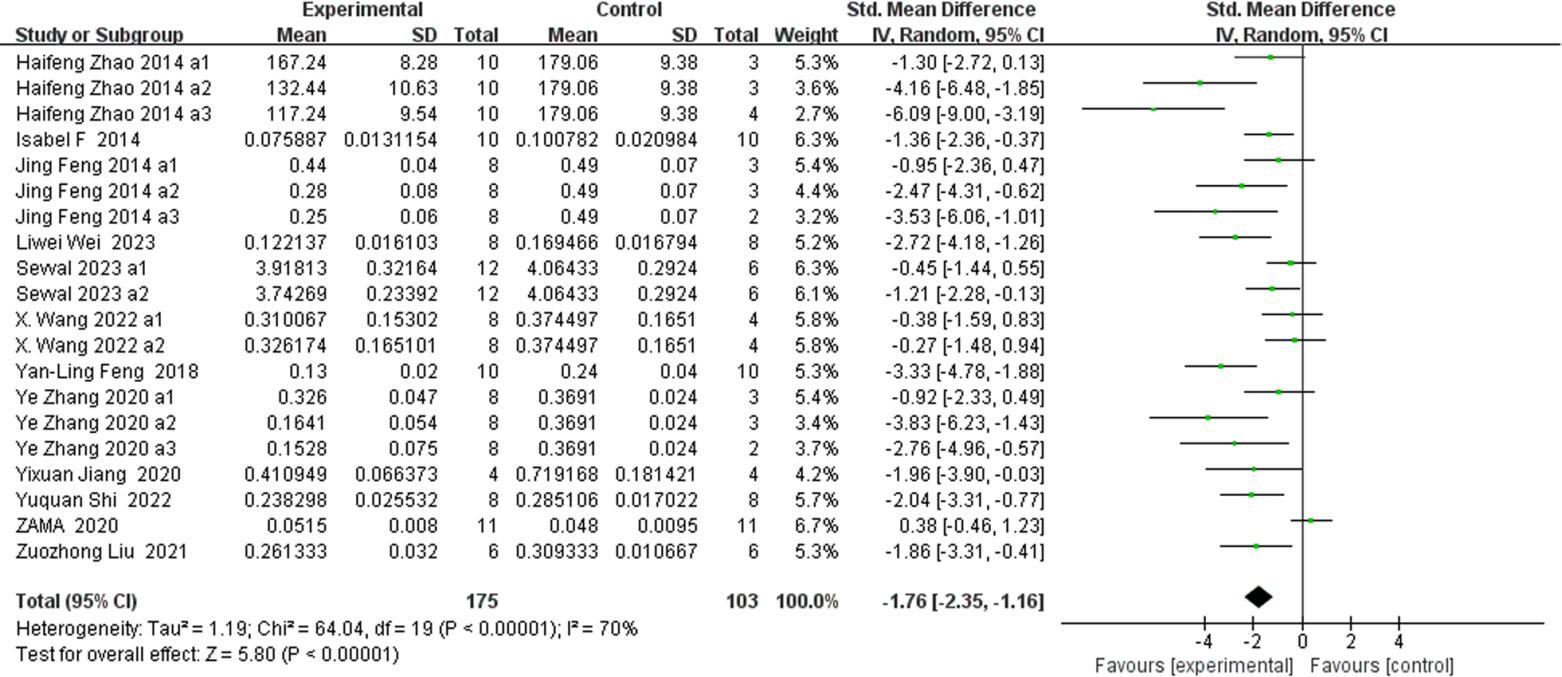
The meta-analysis results of Res for Tb.Sp

1. BV/TV
  Analysis of 18 studies [15, 25–27, 30, 33, 35–38, 40, 41] showed that, compared with those in the control group, the BV/TV in the Res group was markedly greater (*n* = 252; SMD, 1.44; 95% CI, 0.68 to 2.19; *I*^2^ = 80%, *P* < 0.001).
2. Tb.N
  Analysis of 18 studies [19, 25–27, 33, 35–38, 40, 41] showed that, compared with the control group, the Res group had markedly greater total bilirubin (Tb.N) (*n* = 242; SMD, 1.68; 95% CI, 0.95 to 2.42; *I*^2^ = 74%, *P* < 0.00001).
3. Tb.Th
  Analysis of 18 studies [19, 25, 26, 30, 33, 35–37, 40, 41] showed that, compared with that in the control group, the Tb.Th in the Res group was markedly greater (*n* = 248; SMD, 1.73; 95% CI, 1.09 to 2.37; *I*^2^ = 71%, *P* < 0.00001).
4. Tb.Sp
  Analysis of 20 studies [19, 25–27, 30, 33, 35–38, 40, 41] showed that, compared with that in the control group, the Tb.Sp in the Res group was markedly lower (*n* = 278; SMD, − 1.76; 95% CI, − 2.35 to − 1.16; *I*^2^ = 70%, *P* < 0.00001).

### Serum BTM concentrations (Figs. 9, 10, 11, 12, 13 and 14)

**Fig. 9:**
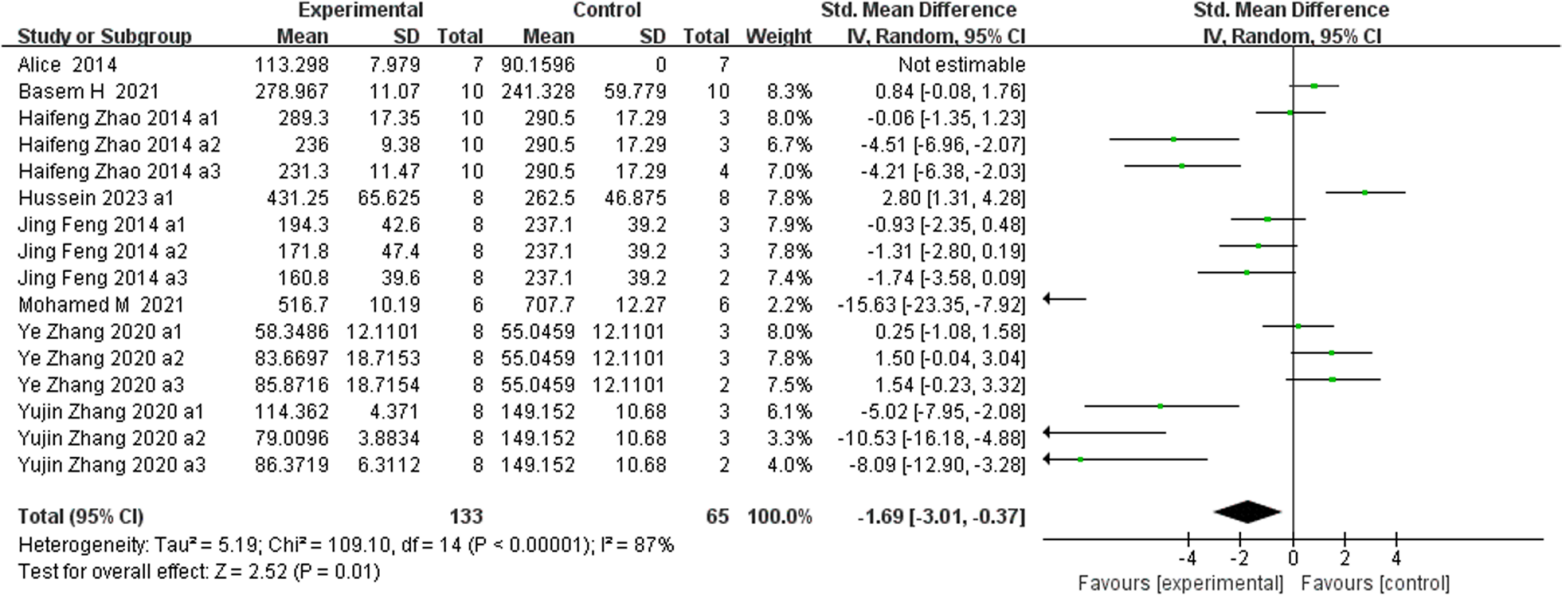
The meta-analysis results of Res for ALP

**Fig. 10:**
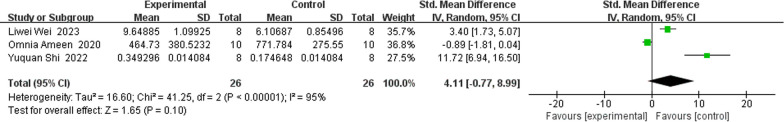
The meta-analysis results of Res for bALP

**Fig. 11:**
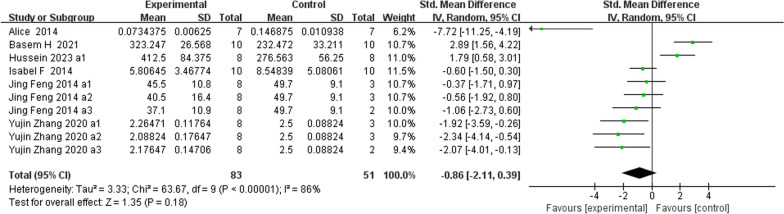
The meta-analysis results of Res for OC

**Fig. 12:**
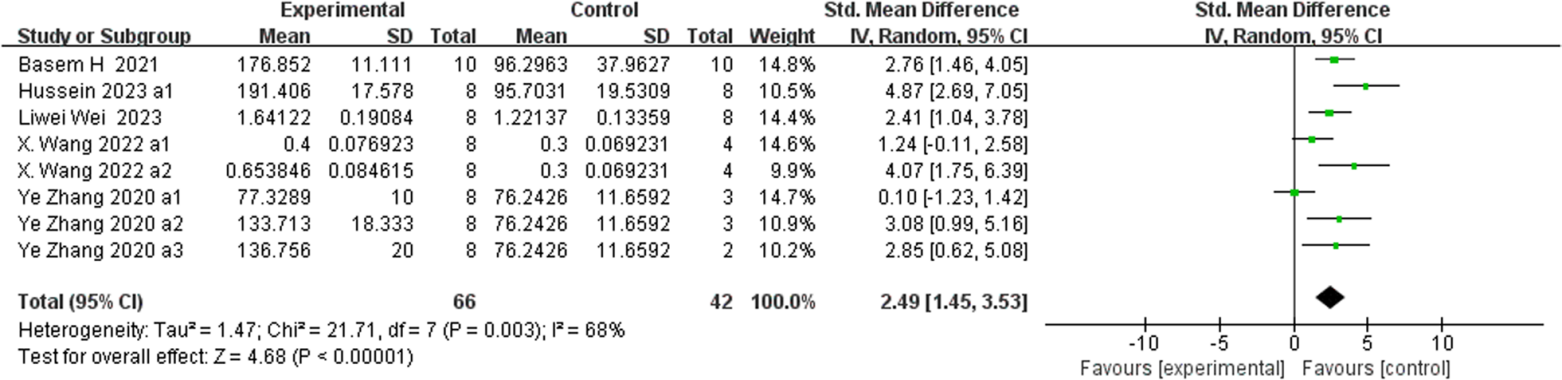
The meta-analysis results of Res for OPG

**Fig. 13:**
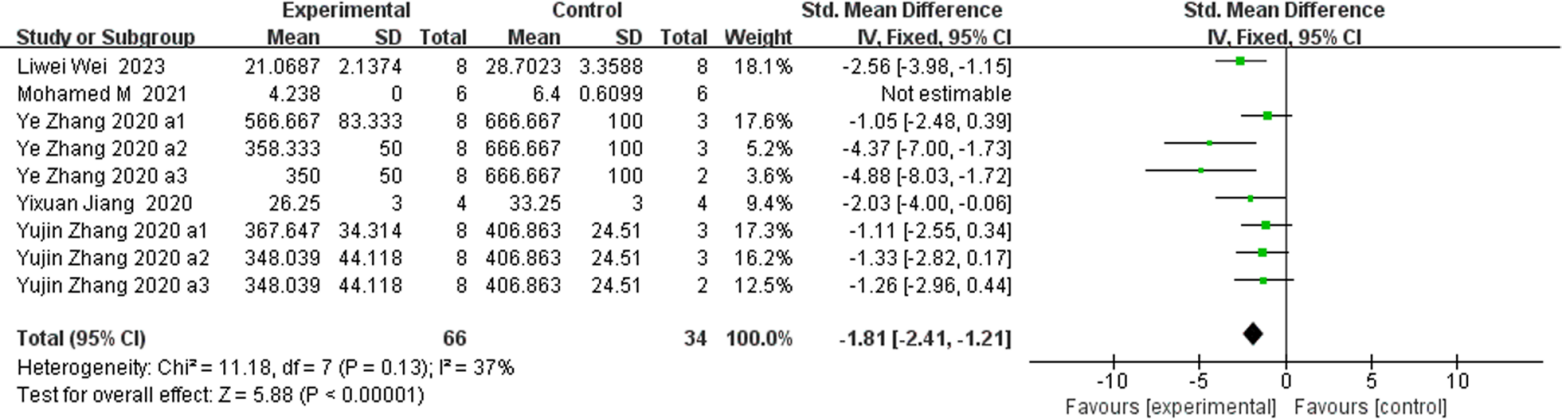
The meta-analysis results of Res for CTX-1

**Fig. 14:**
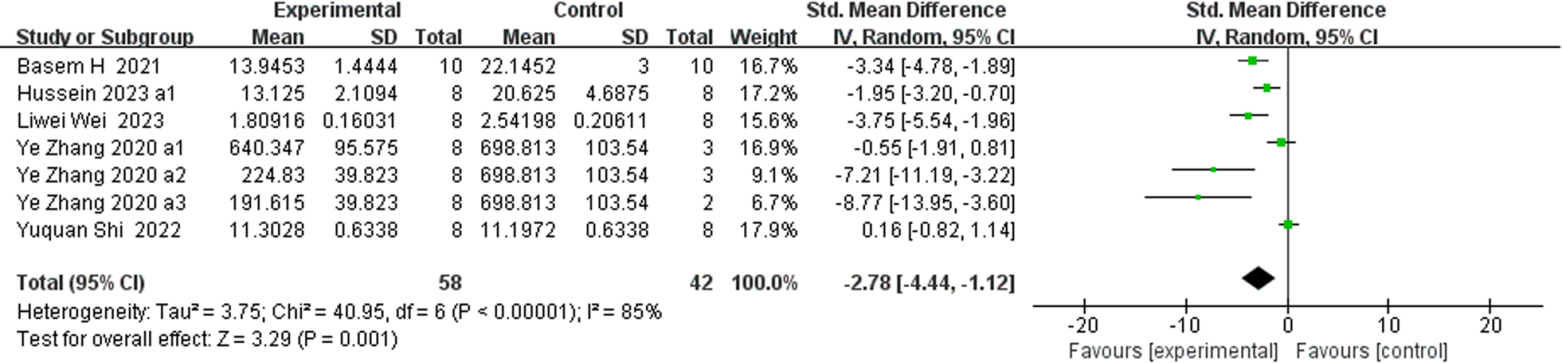
The meta-analysis results of Res for TRAP5b

1. ALP
  Analysis of 16 studies [15, 19, 22, 24, 25, 31, 39, 40] showed that, compared with that in the control group, ALP was markedly lower in the Res group (*n* = 198; SMD, − 1.69; 95% CI, − 3.01 to − 0.37; *I*^2^ = 87%; *P* < 0.05).
2. bALP
  Analysis of 3 studies [21, 36, 37] showed that, compared with those in the control group, the bALP levels in the Res group were markedly greater (*n* = 52; SMD, 4.11; 95% CI, − 0.77 to 8.99; *I*^2^ = 95%, *P* > 0.05).
3. OC
  Analysis of 10 studies [15, 22, 25, 31, 33] showed that, compared with that in the control group, the OC in the Res group was markedly lower (*n* = 134; SMD, − 0.86; 95% CI, − 2.11 to 0.39; *I*^2^ = 86%, *P* > 0.05).
4. Serum OPG
  Analysis of 8 studies [22, 31, 35, 36, 40] showed that, compared with the control group, the Res group had markedly greater OPG levels (*n* = 108; SMD, 2.49; 95% CI, 1.45 to 3.53; *I*^2^ = 68%, *P* < 0.00001).
5. CTX-1
  Analysis of 9 studies [24, 27, 36, 39, 40] showed that, compared with the control group, the Res group had markedly lower CTX-1 levels (*n* = 100; SMD, − 1.81; 95% CI, − 2.41 to − 1.21; *I*^2^ = 37%, *P* < 0.00001).
6. TRAP5b
  Analysis of 7 studies [22, 31, 36, 37, 40] showed that, compared with the control group, the Res group had markedly lower TRAP5b levels (*n* = 100; SMD, − 2.78; 95% CI, − 4.44 to − 1.12; *I*^2^ = 85%, *P* < 0.01).

## Discussion

This review assessed the protective effects of Res in animal models of primary osteoporosis. Twenty-four articles were analyzed, and eleven results were obtained. This review showed that Res can markedly increase BMD, improve morphometric indices of the trabecular microstructure and serum BTMs concentration, and exert a protective effect on animal models of primary OP.

BMD, a gold standard for diagnosing OP, can be detected via dual-energy X-ray (DXA) or micro-CT. Mizutani K et al. reported that Res can alleviate the decrease in femoral BMD induced by ovariectomy in rats [18, 42], while Li YT et al. suggested that inhibiting bone resorption may be related to the ability of Res to increase BMD because Res can inhibit the production of prostaglandin e2 and interleukin-6 [43]. Kenny reported that the serum testosterone concentration is positively correlated with the BMD, and Res may ameliorate bone loss caused by male hypogonadism by maintaining the balance between RANK and OPG [31]. Therefore, our study evaluated the ability of Res to improve BMD in primary OP patients. This study showed that the BMD in the Res group increased significantly. In addition, the subgroup analysis according to the modeling methods, detection methods or detection positions also yielded significant results. In addition, due to the high heritability of BMD, one study had explored the reason why individual differences exist in the effectiveness of bisphosphonates (a first-line anti-OP drug at present) from the genetics. The findings revealed that, in contrast to rs1544410 A/G, another variant, rs2228570 C/T associated with the vitamin D receptor, exhibited a correlation with a favorable response to antiresorptive therapy. This study prompts us to explore whether the genetic characteristics of diverse primary OP conditions influence the efficacy of resveratrol in improving BMD, given its promising results [44].

It is well-known that the morphometric indices of the trabecular microstructure play a vital role in the diagnosis of osteoporosis [45]. The BV/TV is one of the key indices of trabecular microstructure, and an increase in this parameter indicates that bone anabolism is more common than catabolism and that the bone mass is greater, and vice versa. Ozturk S et al. reported that Res (80 mg/kg/day) can reduce the BV/TV, Tb.N and Tb.Th and prevent a sharp decrease in bone mass caused by ovariectomy by improving the microstructure and biophysical and chemical properties of bone [27]. Therefore, our study evaluated the BV/TV in an animal model of primary OP and revealed that Res can improve bone mass and bone metabolism by increasing the BV/TV. In addition, Tb.Th, Tb.N, and Tb.Sp are the primary parameters used to evaluate the spatial morphological structure of trabecular bone. Once osteoporosis occurs, the Tb.Sp increases, while the Tb.N and Tb.Th decrease. Therefore, by evaluating the above three indices, this study revealed that Res can increase the Tb.Th and Tb.N while reducing the Tb.Sp, thus improving bone loss in an animal model of OP.

Intermediate metabolites or enzymes produced during bone turnover are called serum BTMs. BTMs can be classified as bone formation or bone resorption markers. The former indicates osteoblast activity and bone formation, such as ALP, bALP and OC, while the latter reflects osteoclast activity and bone resorption, such as CTX-1 and TRAP5b. BTMs play a role in diagnosing various bone diseases, determining bone turnover types, predicting the risk of fracture, monitoring treatment compliance and evaluating drug efficacy [22, 36, 40]. The level of BTMs in primary OP is usually normal or slightly elevated. Feng J et al. reported that Res inhibits the generation of osteoclasts in OVX rats by decreasing RANKL and TRAP5b and increasing OPG. This difference may be related to the antiapoptotic, antioxidative and anti-inflammatory effects of Res [26]. Elesawy reported that chronic administration of Res can significantly improve the BMD of the tibia, and the protective mechanism may involve increasing the levels of OC, OPG and ALP [22]. Therefore, the present study showed that ALP, CTX-1 and TRAP5b in the Res group were significantly decreased, and OC tended to decrease. In addition, bALP showed an opposite trend to that of the other indicators, which may be due to the small amount of data. OPG is a metabolite secreted by osteoblasts and can inhibit the formation of osteoclasts by competitively binding with RANK [22, 36, 40]. Our results showed that the OPG in the Res group increased significantly, which improved the state of primary OP. In addition to the above markers, there are other bone metabolic intermediates, including p1np, p1cp, LCa/Cr, dpyr, and ntx [45, 46]. Unfortunately, this review did not pursue further investigation due to challenges in acquiring adequate data.

The study limitations were as follows: (1) Language bias; (2) the reliability of the risk of bias assessment was limited to low-quality included studies; and (3) most of the results were highly heterogeneous; however, we performed a subgroup analysis. The results of the sensitivity analysis were robust (Additional file 1).

## Conclusions

Res can markedly increase BMD, improve morphometric indices of the trabecular microstructure and serum BTM concentration, and exert a protective effect on animal models of primary osteoporosis. This study can provide an experimental reference for Res in primary OP. In the future, additional studies are needed to evaluate the effects of Res as an anti-primary OP drug.

### Supplementary Information

**Additional file 1**. PRISMA checklist.

## Data Availability

The datasets used and/or analyzed during the current study are available from the corresponding author on reasonable request.

## Abbreviations

Res: Resveratrol
OP: Osteoporosis
BMD: Bone mineral density
BP: Bisphosphonates
SERM: Estrogen receptor modulators
CI: Confidence interval
SMD: Standardized mean difference
BV/TV: Bone volume/total volume
Tb.N: Trabecular number
Tb.Th: Trabecular thickness
Tb.Sp: Trabecular separation
OC: Osteocalcin
ALP: Alkaline phosphatase
OPG: Serum osteoprotegerin
bALP: Bone alkaline phosphatase
CTX-1: Type I collagen strong carboxyl peptide
TRAP5b: Tartrate-resistant acid phosphatase 5b
BTMs: Bone turnover markers
OVX: Ovariectomized
SEM: Standard error of the mean
SD: Standard deviation
