# Supplementary material for: The effects of resveratrol in animal models of primary osteoporosis: a systematic review and meta-analysis
Source: J Orthop Surg Res. 2024 Feb 13;19:137. doi: 10.1186/s13018-024-04595-1 (PMC10863264; doi:10.1186/s13018-024-04595-1)
Supplement: Supplementary file 1 — Additional file 1. PRISMA checklist. [file 13018_2024_4595_MOESM1_ESM.docx]

**Part 1. The funnel plot**


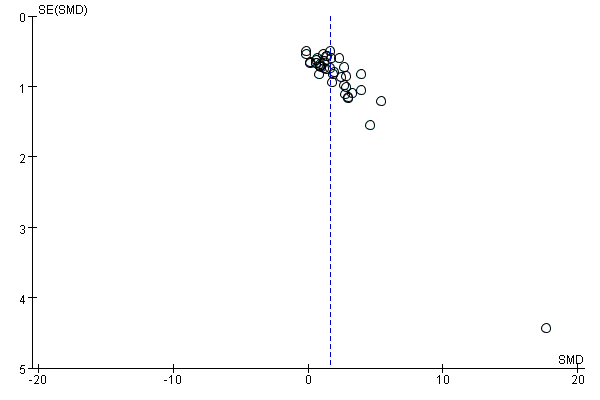


**Figure 1.** The funnel plot for BMD.


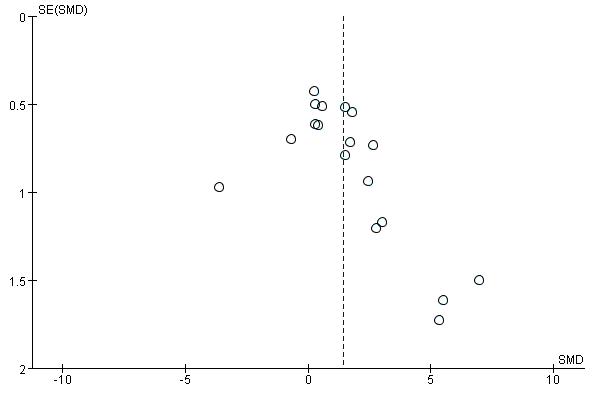


**Figure 2.** The funnel plot for **BV/TV**.


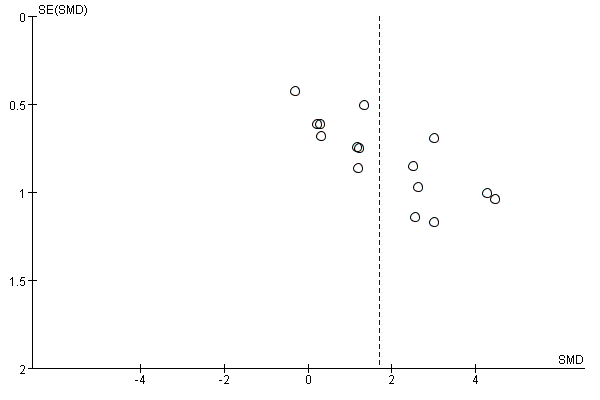


**Figure 3.** The funnel plot for **Tb.N**.


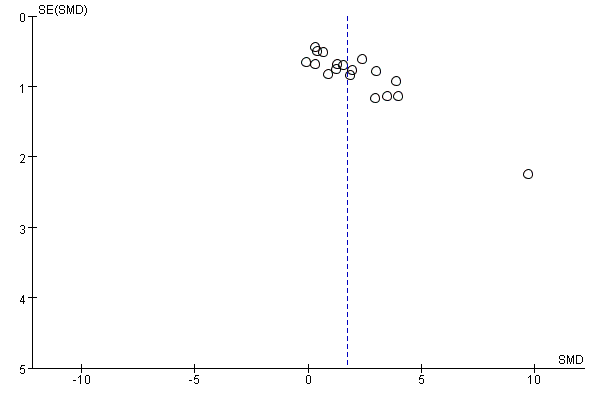


**Figure 4.** The funnel plot for **Tb.Th**.


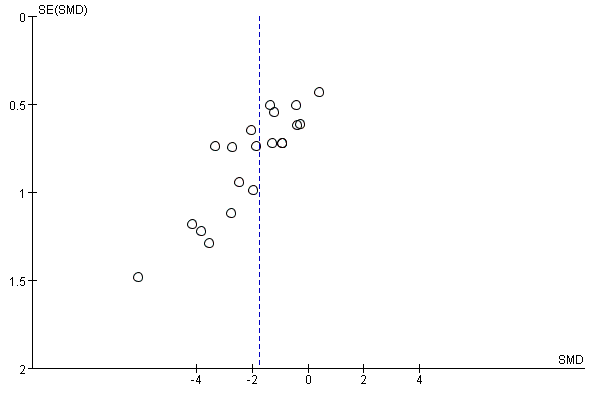


**Figure 5.** The funnel plot for **Tb.Sp**.


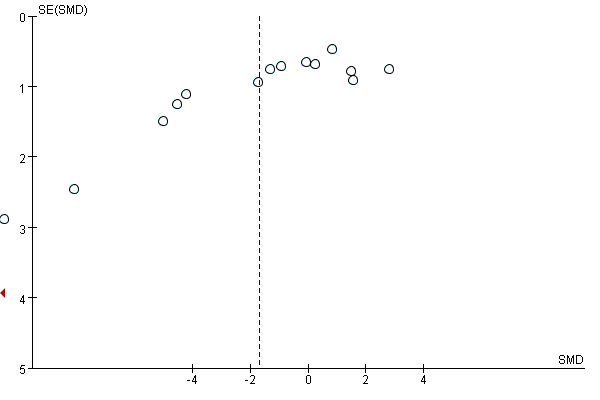


**Figure 6.** The funnel plot for **ALP**.


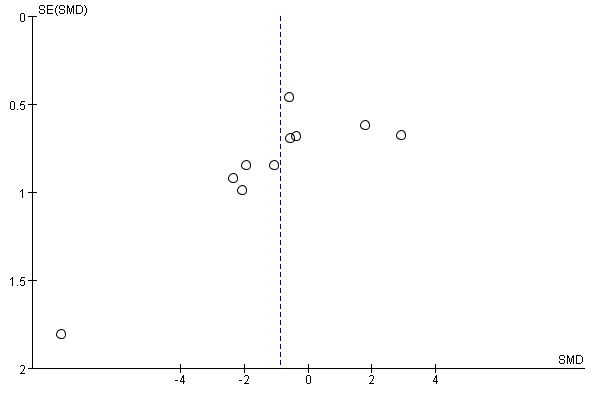


**Figure 7.** The funnel plot for OC.

**Part 2.sensitivity analysis**

**
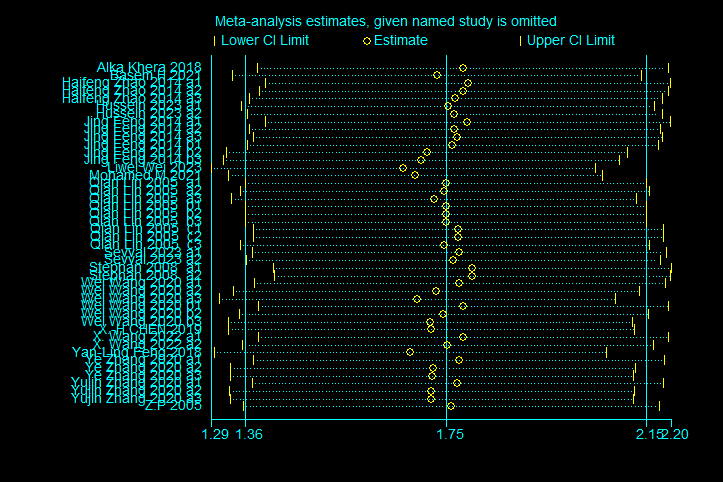
**

**Figure 8.Sensitivity analysis for BMD.**

**
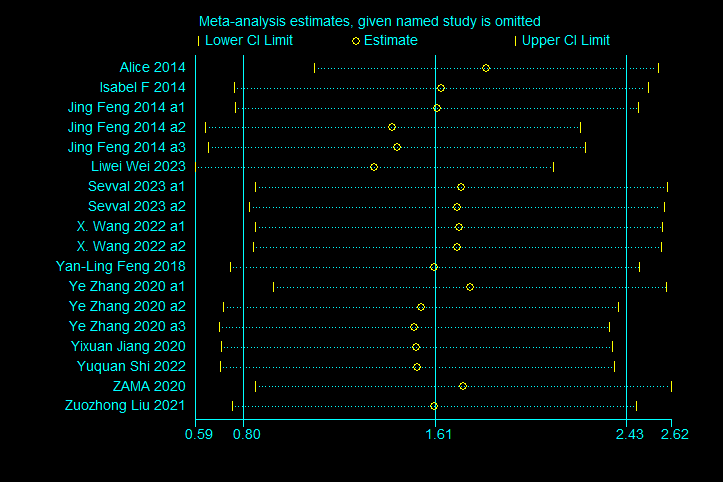
**

**Figure 9.Sensitivity analysis for BV/TV.**

**
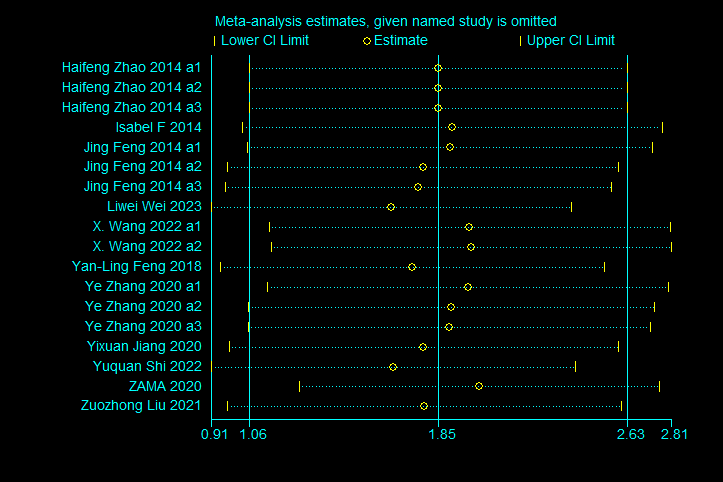
**

**Figure 10.Sensitivity analysis for Tb.N.**

**
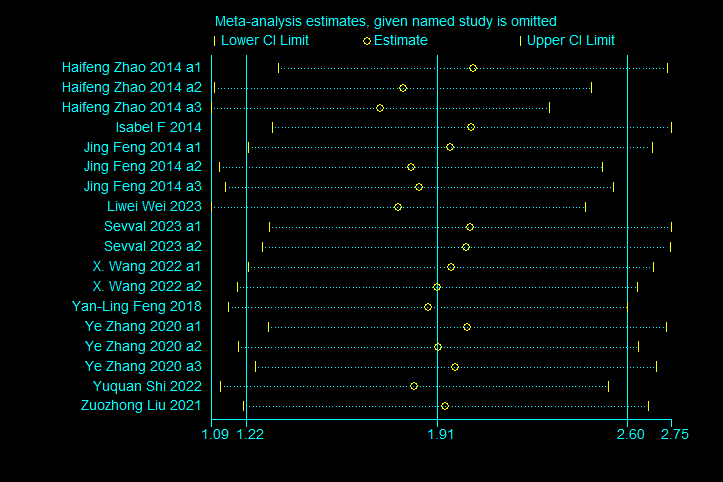
**

**Figure 11.Sensitivity analysis for Tb.Th.**

**
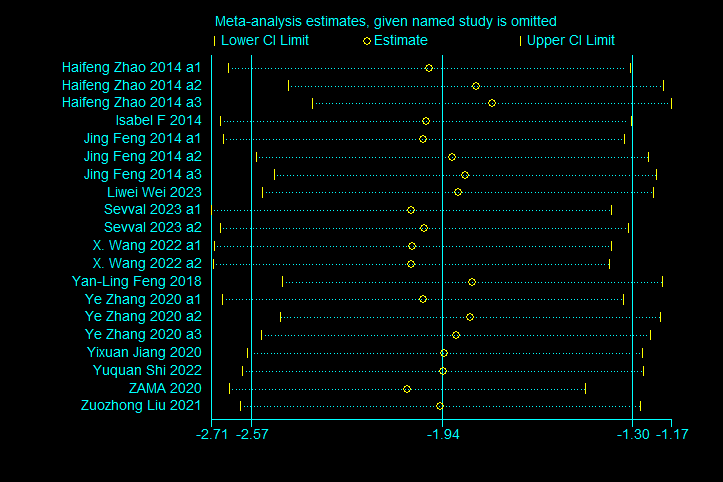
**

**Figure 12.Sensitivity analysis for Tb.Sp.**

**
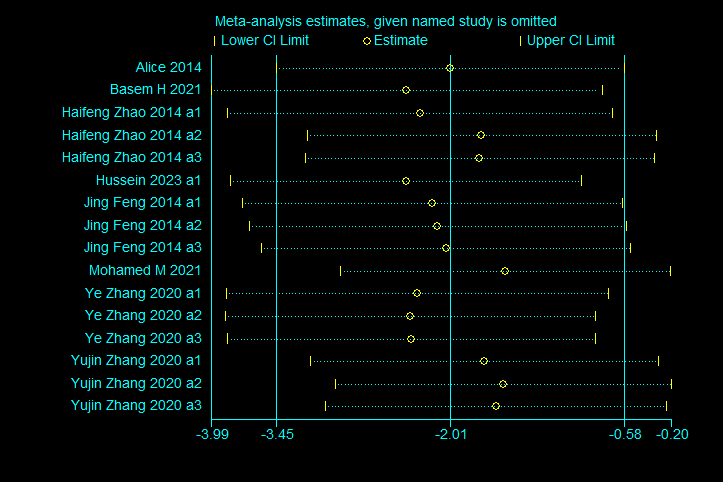
**

**Figure 13.Sensitivity analysis for ALP.**

**
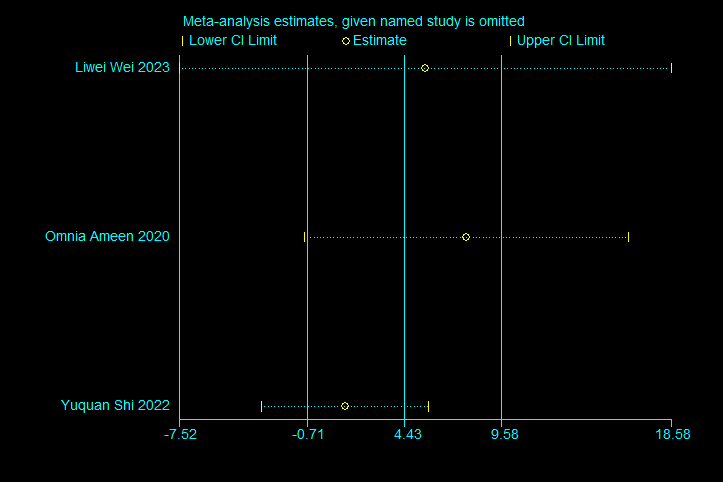
**

**Figure 14.Sensitivity analysis for bALP.**

**
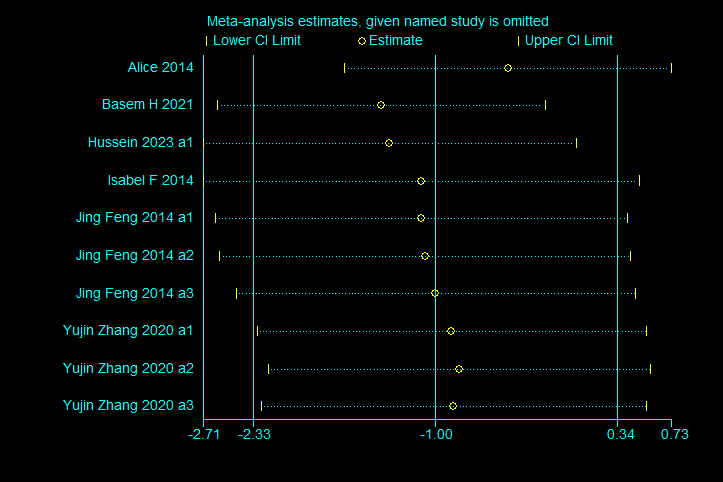
**

**Figure 15.Sensitivity analysis for OC.**

**
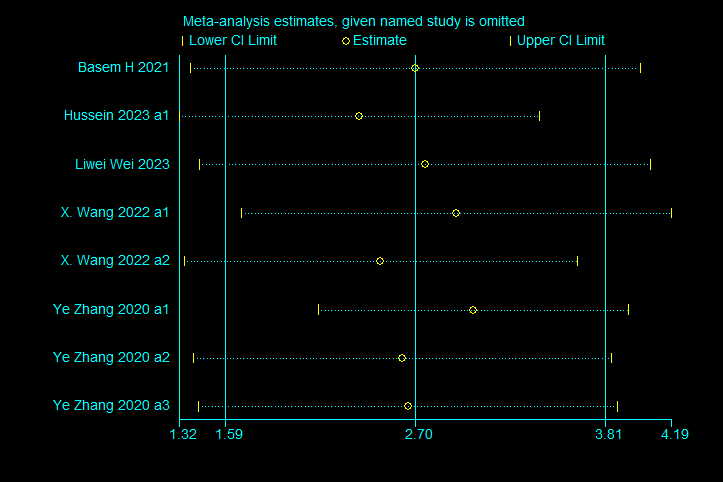
**

**Figure 16.Sensitivity analysis for OPG.**

**
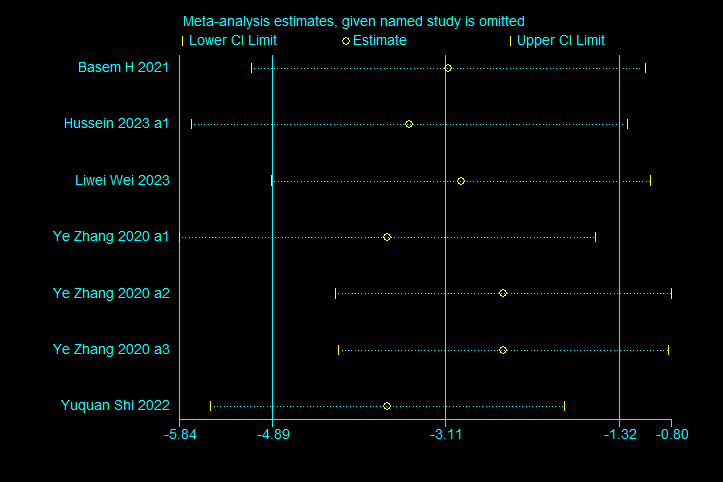
**

**Figure 17.Sensitivity analysis for TRAP5b.**

**Part 3. Search strategies**

**Pubmed**

**(("Resveratrol"[Mesh]) OR ((((((((((((((((Resveratrol[Title/Abstract]) OR (3,5,4'-Trihydroxystilbene[Title/Abstract])) OR (3,4',5-Trihydroxystilbene[Title/Abstract])) OR (3,4',5-Stilbenetriol[Title/Abstract])) OR (trans-Resveratrol-3-O-sulfate[Title/Abstract])) OR (trans Resveratrol 3 O sulfate[Title/Abstract])) OR (SRT 501[Title/Abstract])) OR (SRT501[Title/Abstract])) OR (SRT-501[Title/Abstract])) OR (cis-Resveratrol[Title/Abstract])) OR (cis Resveratrol[Title/Abstract])) OR (Resveratrol, (Z)-[Title/Abstract])) OR (trans-Resveratrol[Title/Abstract])) OR (trans Resveratrol[Title/Abstract])) OR (Resveratrol-3-sulfate[Title/Abstract])) OR (Resveratrol 3 sulfate[Title/Abstract]))) AND (((((((((((((((((((((((Osteoporosis[Title/Abstract]) OR (Osteoporoses[Title/Abstract])) OR (Osteoporosis, Post-Traumatic[Title/Abstract])) OR (Osteoporosis, Post Traumatic[Title/Abstract])) OR (Post-Traumatic Osteoporoses[Title/Abstract])) OR (Post-Traumatic Osteoporosis[Title/Abstract])) OR (Osteoporosis, Senile[Title/Abstract])) OR (Osteoporoses, Senile[Title/Abstract])) OR (Senile Osteoporoses[Title/Abstract])) OR (Osteoporosis, Involutional[Title/Abstract])) OR (Senile Osteoporosis[Title/Abstract])) OR (Osteoporosis, Age-Related[Title/Abstract])) OR (Osteoporosis, Age Related[Title/Abstract])) OR (Bone Loss, Age-Related[Title/Abstract])) OR (Age-Related Bone Loss[Title/Abstract])) OR (Age-Related Bone Losses[Title/Abstract])) OR (Bone Loss, Age Related[Title/Abstract])) OR (Osteoporoses, Age-Related[Title/Abstract])) OR (Bone Losses, Age-Related[Title/Abstract])) OR (Age-Related Osteoporoses[Title/Abstract])) OR (Age Related Osteoporosis[Title/Abstract])) OR (Age-Related Osteoporosis[Title/Abstract])) OR ("Osteoporosis"[Mesh]))**

****Cochrane****

**#1 MeSH descriptor: [Resveratrol] explode all trees**

**#2 (Resveratrol):ti,ab,kw OR (trans Resveratrol):ti,ab,kw OR (trans-Resveratrol):ti,ab,kw OR (cis-Resveratrol):ti,ab,kw OR (cis Resveratrol):ti,ab,kw**

**#3 (trans Resveratrol 3 O sulfate):ti,ab,kw OR (SRT501):ti,ab,kw OR (SRT-501):ti,ab,kw OR (SRT 501):ti,ab,kw OR (Resveratrol 3 sulfate):ti,ab,kw**

**#4 #1 or #2 or #3**

**#5 MeSH descriptor: [Osteoporosis] explode all trees**

**#6 (Osteoporosis):ti,ab,kw OR (Osteoporoses, Senile):ti,ab,kw OR (Osteoporosis, Involutional):ti,ab,kw OR (Senile Osteoporosis):ti,ab,kw OR (Senile Osteoporoses):ti,ab,kw**

**#7 (Osteoporosis, Senile):ti,ab,kw OR (Osteoporosis, Age-Related):ti,ab,kw OR (Age-Related Osteoporosis):ti,ab,kw OR (Osteoporosis, Age Related):ti,ab,kw OR (Osteoporoses, Age-Related):ti,ab,kw**

**#8 (Bone Losses, Age-Related):ti,ab,kw OR (Bone Loss, Age-Related):ti,ab,kw OR (Age-Related Bone Loss):ti,ab,kw OR (Age-Related Osteoporoses):ti,ab,kw OR (Age-Related Bone Losses):ti,ab,kw**

**#9 (Bone Loss, Age Related):ti,ab,kw OR (Age Related Osteoporosis):ti,ab,kw OR (Osteoporoses):ti,ab,kw OR (Post-Traumatic Osteoporosis):ti,ab,kw OR (Osteoporosis, Post Traumatic):ti,ab,kw**

**#10 (Post-Traumatic Osteoporoses):ti,ab,kw OR (Osteoporosis, Post-Traumatic):ti,ab,kw**

**#11 #5 or #6 or #7 or #8 or #9 or #10**

**#12 #4 and #11**

****Embase****

**#9. ('resveratrol'/exp OR ('3, 4`, 5**

**stilbenetriol':ab,ti OR '3, 4`, 5**

**trihydroxystilbene':ab,ti OR '5 (4 hydroxystyryl)**

**benzene 1, 3 diol':ab,ti OR 'srt 501':ab,ti OR**

**'srt501':ab,ti OR 'trans resveratrol':ab,ti OR**

**'trans-resveratrol':ab,ti OR**

**'resveratrol':ab,ti)) AND ('osteoporosis'/exp OR**

**('decalcification, pathologic':ab,ti OR**

**'endocrine osteoporosis':ab,ti OR 'osteoporotic**

**decalcification':ab,ti OR 'pathologic**

**decalcification':ab,ti OR 'osteoporosis':ab,ti))**

**#8. 'osteoporosis'/exp OR ('decalcification,**

**pathologic':ab,ti OR 'endocrine**

**osteoporosis':ab,ti OR 'osteoporotic**

**decalcification':ab,ti OR 'pathologic**

**decalcification':ab,ti OR 'osteoporosis':ab,ti)**

**#7. 'resveratrol'/exp OR ('3, 4`, 5**

**stilbenetriol':ab,ti OR '3, 4`, 5**

**trihydroxystilbene':ab,ti OR '5 (4 hydroxystyryl)**

**benzene 1, 3 diol':ab,ti OR 'srt 501':ab,ti OR**

**'srt501':ab,ti OR 'trans resveratrol':ab,ti OR**

**'trans-resveratrol':ab,ti OR 'resveratrol':ab,ti)**

**#6. 'decalcification, pathologic':ab,ti OR 'endocrine**

**osteoporosis':ab,ti OR 'osteoporotic**

**decalcification':ab,ti OR 'pathologic**

**decalcification':ab,ti OR 'osteoporosis':ab,ti**

**#5. '3, 4`, 5 stilbenetriol':ab,ti OR '3, 4`, 5**

**trihydroxystilbene':ab,ti OR '5 (4 hydroxystyryl)**

**benzene 1, 3 diol':ab,ti OR 'srt 501':ab,ti OR**

**'srt501':ab,ti OR 'trans resveratrol':ab,ti OR**

**'trans-resveratrol':ab,ti OR 'resveratrol':ab,ti**

**#4. 'decalcification, pathologic' OR 'endocrine**

**osteoporosis' OR 'osteoporotic decalcification'**

**OR 'pathologic decalcification' OR 'osteoporosis'**

**#3. 'osteoporosis'/exp**

**#2. '3, 4`, 5 stilbenetriol' OR '3, 4`, 5**

**trihydroxystilbene' OR '5 (4 hydroxystyryl)**

**benzene 1, 3 diol' OR 'srt 501' OR 'srt501' OR**

**'trans resveratrol' OR 'trans-resveratrol' OR**

**'resveratrol'**

**#1. 'resveratrol'/exp**

**Web of science**

**#5 #4 AND #3**

**#4 #2 OR #1**

**#3 ((((((((((((((((((((((TS=(Osteoporosis)) OR ALL=(Osteoporosis)) OR ALL=(Osteoporoses, Age-Related)) OR ALL=(Age-Related Osteoporoses )) OR ALL=(Age Related Osteoporosis )) OR ALL=(Age-Related Osteoporosis )) OR ALL=(Bone Losses, Age-Related )) OR ALL=(Bone Loss, Age Related )) OR ALL=(Age-Related Bone Losses )) OR ALL=(Age-Related Bone Loss )) OR ALL=(Bone Loss, Age-Related )) OR ALL=(Osteoporosis, Age Related )) OR ALL=(Osteoporosis, Age-Related )) OR ALL=(Senile Osteoporosis )) OR ALL=(Osteoporosis, Involutional )) OR ALL=(Senile Osteoporoses )) OR ALL=(Osteoporoses, Senile )) OR ALL=(Osteoporosis, Senile )) OR ALL=(Post-Traumatic Osteoporosis )) OR ALL=(Post-Traumatic Osteoporoses )) OR ALL=(Osteoporosis, Post Traumatic )) OR ALL=(Osteoporosis, Post-Traumatic )) OR ALL=(Osteoporoses )**

**#2 (((((((((((((((ALL=(3,5,4'-Trihydroxystilbene)) OR ALL=(3,4',5-Trihydroxystilbene)) OR ALL=(3,4',5-Stilbenetriol)) OR ALL=(trans-Resveratrol-3-O-sulfate)) OR ALL=(trans Resveratrol 3 O sulfate)) OR ALL=(SRT-501)) OR ALL=(SRT501 )) OR ALL=(SRT 501 )) OR ALL=(Resveratrol, (Z)-)) OR ALL=(cis Resveratrol )) OR ALL=(cis-Resveratrol )) OR ALL=(Resveratrol 3 sulfate)) OR ALL=(Resveratrol-3-sulfate )) OR ALL=(trans Resveratrol )) OR ALL=(trans-Resveratrol )) OR ALL=(Resveratrol)**

**#1 TS=(resveratrol)**
